# Prolactin related symptoms during risperidone maintenance treatment: results from a prospective, multicenter study of schizophrenia

**DOI:** 10.1186/s12888-016-1103-3

**Published:** 2016-11-09

**Authors:** Qijing Bo, Fang Dong, Xianbin Li, Zhimin Wang, Xin Ma, Chuanyue Wang

**Affiliations:** 1 Beijing Key Laboratory of Mental Disorders, Department of Psychiatry, Beijing Anding Hospital, Capital Medical University, No.5 Ankang Lane, Dewai Avenue, Xicheng District, Beijing 100088 China; 2 Center of Schizophrenia, Beijing Institute for Brain Disorders, Laboratory of Brain Disorders (Capital Medical University), Ministry of Science and Technology, Beijing, 100088 China

**Keywords:** Side effects, Prolactin related symptoms, Schizophrenia, Maintenance treatment, Antipsychotics, Risperidone, Generalized linear mixed model (GLMM)

## Abstract

**Background:**

This study aimed to investigate prolactin related symptoms (PRS) in individuals with schizophrenia during risperidone maintenance treatment for one year, as well as to identify the risk factors for PRS.

**Methods:**

In a multicenter, randomized, controlled, longitudinal study, clinically stabilized schizophrenia patients (*N* = 374) were randomized to a no-dose-reduction group (*N* = 129) and 4-week (*N* = 125) and 26-week (*N* = 120) reduction groups, in which the original dose was followed by a 50 % reduction over 8 weeks and subsequently maintained. PRS were assessed via a scale of prolactin related adverse events, which included 16 items: menstrual cycle, menstrual period, menstrual volume, menstrual irregularities, amenorrhea, dysmenorrhea, postpartum lactation, gynecomastia, breast tenderness, sexual dysfunction, decreased sexual desire, erectile dysfunction, ejaculatory dysfunction, impotence, increased body hair, and acne. The occurrence of PRS was assessed at baseline and monthly for six months, followed by every two months. A mixed model was used.

**Results:**

PRS at baseline were reported in 18.4, 15.0, and 14.0 % of the 4-week, 26-week, and no-dose-reduction groups, respectively. Female gender, younger age at onset, and the Positive and Negative Syndrome Scale (PANSS) total scores at entry predicted the development of PRS. The mixed model indicated that PRS were more severe in females and at a high dose. In the 237 patients who remained in the study after one year, the incidence of PRS decreased to 9.6, 11.1, and 7.6 % in the 4-week, 26-week, and no-dose-reduction groups, respectively.

**Conclusion:**

These findings indicate that the PRS severity was alleviated during the one year treatment period because of the dose reduction. Attention should focus on the side effects of hyperprolactinemia during long-term treatment, especially with a high dose, females, younger age at onset, and more severe patients.

**Trial registration:**

ClinicalTrials.gov identifier: NCT00848432. Registered February 19, 2009.

## Background

Hyperprolactinemia is one of the most common antipsychotic-induced adverse events in psychiatric patients [1], especially patients treated with first generation antipsychotics or the second generation antipsychotics risperidone or amisulpride [2–4]. In Chinese schizophrenia patients, the hyperprolactinemia rate was increased in patients treated with risperidone compared with quetiapine, olanzapine, clozapine and aripiprazole [5, 6]. Moreover, the prevalence of hyperprolactinemia in females treated with risperidone was greater than conventional antipsychotic medications [7]. A substantial number of studies have demonstrated that the treatment of schizophrenia with risperidone may cause a substantial plasma prolactin increase and an unacceptably high incidence of prolactin related symptoms (PRS), such as amenorrhea, galactorrhea, gynecomastia, and sexual dysfunction [8, 9].

PRS, including menstrual disturbances, amenorrhea, galactorrhea, sexual dysfunction, gynecomastia, and impotence [10–12], may lead to clinical consequences, such as infertility [9], decreased bone mineral density and fracture risk [13, 14], metabolic syndrome [15], exacerbation of autoimmune disorders [16], risks for cardiovascular disease [17], breast or prostate cancers [18, 19], poor treatment adherence, and fluctuations in psychotic symptoms [20]. These associated consequences may seriously affect patient quality of life. Specifically, the treatment of schizophrenia with risperidone may cause a substantial prolactin increase and an unacceptably high incidence of PRS, such as amenorrhea, galactorrhea, gynecomastia, and sexual dysfunction [3, 8, 9]. Thus, increased attention should focus on the PRS of risperidone, which is widely used in schizophrenia treatment.

The risperidone maintenance treatment in schizophrenia (RMTS) study was designed to determine the duration of maintenance treatment required with the initial therapeutic dose compared with a reduced dose over time. The main results of the study were published in 2010 [21], and specific issues, such as sex differences [22], cigarette smoking [22], weight changes [23], socio-demographic and clinical profiles of paranoid and nonparanoid schizophrenia [24], predictors of relapse [25], and extrapyramidal symptoms [26], have been addressed in subsequent papers. Regarding PRS, the initial analysis analyzed the presence of prolactin-related adverse events at 4 weeks and the end of the study, regardless of the other time points.

This study aimed to investigate the PRS of risperidone maintenance treatment in schizophrenia. The first objective was to investigate the baseline PRS associated with risperidone and the socio-demographic and clinical predictors. The second objective was to compare the trajectory of PRS over time in different dose groups, as well as identify potentially related factors.

## Methods

### Participants

Complete baseline data for PRS were available for 374 participants, which comprised 46 % males and 54 % females, with a mean age of 32.6 years (SD = 10.8). Patients were recruited from December 1, 2002 to January 31, 2005 at 19 mental health centers in China, which represent a range of clinical settings.

The inclusion criteria were as follows: an age between 18 and 65 years; male or female; a diagnosis of DSM-IV schizophrenia; clinical stability defined as <36 points on the Brief Psychiatric Rating Scale (BPRS) [27] for at least 4 weeks but not more than 8 weeks following an acute episode; monotherapy of risperidone at an optimal therapeutic dose (4–8 mg/day) in the acute phase of treatment and had responded to antipsychotic treatment; satisfactory adherence defined by a pill count that yielded more than 80 % adherence to the risperidone prescription over the previous 4 weeks; and ability and willingness to provide written informed consent. The exclusion criteria included a history of or an ongoing major chronic medical or neurological condition; a history of ECT or use of antidepressants, mood stabilizers, or Chinese herbal remedies concomitantly with risperidone or previous participation in any other drug trial or interventional study over the 4 weeks prior to study entry; abuse of drugs or alcohol other than nicotine; and pregnancy or plans to become pregnant, lactation, or lack of an effective method of birth control.

### Design

A computer-based central telephone randomization system was used. The patients who met the inclusion criteria were randomly assigned to three groups: the 4-week group (initial optimal therapeutic dose continued for 4 weeks, followed by a 50 % dose reduction over the subsequent 8 weeks, which was then maintained until the end of the study), the 26-week group (initial optimal therapeutic dose continued for 26 weeks, followed by a 50 % dose reduction over 8 weeks until the end of the study), and the no-dose-reduction group (initial optimal therapeutic dose continued throughout the study). Dose adjustments were not allowed prior to the dose reduction period. The study continued until the last recruited patient completed his or her one-year follow-up. Patients were excluded from the study because of several conditions: relapse, pregnancy, a severe medical condition, or newly emerging and intolerable side effects; these patients were subsequently treated as clinically appropriate. The study protocols were approved by the clinical research ethics committees of the respective study centers. Written informed consent was obtained from each participant. The report of the study adheres to the CONSORT guidelines.

### Assessments

Basic socio-demographic and clinical characteristics were collected using a questionnaire designed for the study. The BPRS was used as a screening tool at entry, which was used to measure psychiatric symptoms. Relapse was defined according to Csernansky’s criteria [28]. Psychopathology was evaluated using the Chinese version of the PANSS [29]. PRS were assessed via a scale of prolactin related adverse events, which included 16 items: menstrual cycle, menstrual period, menstrual volume, menstrual irregularities, amenorrhea, dysmenorrhea, postpartum lactation, gynecomastia, breast tenderness, sexual dysfunction, decreased sexual desire, erectile dysfunction, ejaculatory dysfunction, impotence, increased body hair, and acne. For each item, 1 represents yes and 0 represents no symptom. The total score indicates the severity of the PRS. These assessments were performed monthly during the first six months, followed by every two months until the last enrolled patient completed the study, which included the end of the study or the time of relapse, discontinuation, or dropout. All raters comprised qualified psychiatrists who were trained to use the scales prior to the start of the study.

### Statistical analyses

All analyses were conducted with Statistics Analysis System (SAS Institute, Inc., Cary, N.C., USA). At baseline, PRS were described by a binary variable that depended on the prolactin related adverse events. Specifically, the patients were considered to have developed PRS if one of the 16 items was positive (PRS group) at baseline, whereas the remaining patients were considered to have no PRS (No-PRS group). A stepwise logistic regression analysis was used to adjust for relevant covariates and to determine the independent risk factors for PRS. The dependent variable comprised the development of PRS, and the independent variables included age, sex, education, age at onset, duration of illness, risperidone dose at baseline, overall length of risperidone treatment in this episode, length of risperidone treatment at the optimal therapeutic dose, and the total PANSS score.

Comparisons between the groups regarding socio-demographic and clinical characteristics were performed with standard descriptive statistics: analyses of variance (ANOVAs) or non-parametric tests, such as Mann-Whitney U or Kruskal-Wallis tests, for continuous variables. Pearson’s correlations, Chi-square tests, or Fisher’s exact tests were used for categorical variables. To identify the between-group differences, a Hochberg adjustment for multiple comparisons was implemented [30]. To examine the evolution of PRS in the three study groups, a mixed model was estimated, which included fixed covariates for individual background variables, baseline values, time expressed in weeks, the interaction between study groups, and time. The PANSS scores and risperidone dose were added to the model as time-varying covariates. The model distribution of the response variable was continuous with a total score of PRS. All statistical tests were set at 0.05 two-tailed.

## Results

### Baseline PRS and related factors

Table 1 indicates the participants’ socio-demographic and clinical characteristics. There were significantly more males in the PRS group compared with the no-PRS group (*χ*2 = 36.185, *P* < 0.001). Moreover, the PANSS total score was significantly increased in the PRS group compared with the NO-PRS group (*F* = 7.66, *P* = 0.006). There were no significant differences between the groups regarding age, marital status, education level, employment, residence, history of psychiatric disorder(s), age at onset, duration of illness, overall length of risperidone treatment in this episode and at optimal therapeutic doses, or the risperidone doses at baseline. In the stepwise logistic regression analyses, gender, age at onset, and PANSS total scores at entry independently predicted the development of PRS (Table 2).

**Table 1:** Demographics and baseline characteristics

Characteristics	No-PRS group (*N* = 315)		PRS group (*N* = 59)		Statistics		Total (*N* = 374)	
	Mean	SD	Mean	SD	F	P	Mean	SD
Age	32.9	10.9	31.2	10.0	1.30	0.256	32.6	10.8
Education (years)	12.2	2.3	12.6	2.2	1.74	0.188	12.2	2.3
Age at onset	26.6	8.9	24.4	7.6	3.18	0.075	26.3	8.8
Duration of illness (years)	6.6	6.7	7.1	7.7	0.23	0.628	6.7	6.9
Risperidone dose at baseline (mg/d)	4.3	0.6	4.4	0.6	0.30	0.582	4.4	0.6
Total treatment in this episode (months)	5.3	5.1	5.2	4.3	0.04	0.850	5.3	5.0
Overall length of risperidone treatment in this episode (days)	83.5	54.1	90.2	59.7	0.74	0.389	84.5	55.0
Length of risperidone treatment at optimal therapeutic dose (days)	55.4	48.7	54.6	25.1	0.02	0.901	55.2	45.8
PANSS total score	39.1	9.5	42.8	10.0	7.66	0.006	39.5	9.6
	N	%	N	%	*χ*2	P	N	%
Number of male participants	166	52.7	6	10.2	36.185	<0.001	172	46.0
Married	133	42.2	27	45.8	0.440	0.802	160	42.8
Family history of psychiatric disorder(s)	25	7.9	8	13.6	1.953	0.162	33	8.8
Unemployed	149	47.9	30	50.2	1.450	0.484	179	47.8

*PANSS* Positive and Negative Syndrome Scale, *SD* standard deviation

**Table 2:** Independent predictors of prolactin related symptoms (logistic regression model)

	Wald Chi-square	P-value	Odds ratio estimates	95 % C.I.
Gender	31.3	<0.001	13.609	5.452–33.970
Age at onset	5.2	0.022	0.959	0.925–0.994
PANSS total scores	11.4	0.001	1.053	1.022–1.086

*PANSS* Positive and Negative Syndrome Scale, *C.I.* confidence interval

### PRS during one-year treatment

**Fig. 1:**
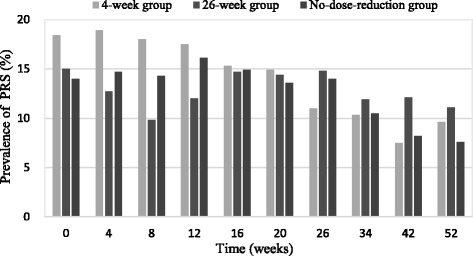
Prevalence of PRS in patients who remained in treatment

The incidences of PRS at baseline and during the 52-week study duration are indicated in Fig. 1. PRS at baseline occurred in 18.4, 15.0, and 14 % of the 4-week, 26-week, and no-dose-reduction groups, respectively. The percentages of PRS for the patients who continued treatment after one year were decreased to 9.6, 11.1, and 7.6 % for the 4-week, 26-week, and no-dose-reduction groups, respectively.

The mixed model indicated there was significant variation in the PRS over time, which were relieved after baseline in all groups (*F* = 6.28, *P* = 0.013). An analysis of the course of PRS using the mixed procedure indicated there was no significant variation in the three groups over time (*F* = 0.17, *P* = 0.684). Risperidone dose as a time-varying covariate was included in the model, and a lower dose indicated less severe PRS (*F* = 18.84, *P* < 0.001). When corrected for the risperidone dose, the effect of time disappeared, which suggests that the variation in the PRS over time was because of the risperidone dose. Gender was entered in the model, and the PRS were more severe in females (*F* = 42.88, *P* < 0.001).

### Dose and medication adherence

Pill counts were used to measure treatment adherence; the percentages of pills taken were 97, 92, and 95 % in the 4-week, 26-week, and no-dose-reduction groups, respectively. Thus, medication adherence was deemed acceptable, and changes in the risperidone dosage are presented in Table 3.

**Table 3:** Changes in risperidone doses

Group	Baseline		4 weeks		12 weeks		20 weeks		26 weeks		34 weeks		52 weeks	
(mg/d)	Mean	SD	Mean	SD	Mean	SD	Mean	SD	Mean	SD	Mean	SD	Mean	SD
4-week dose	4.4	0.8	4.2	0.8	2.2^a^	0.4	2.2	0.5	2.1	0.4	2.1	0.4	2.1	0.4
26-week dose	4.2	0.5	4.2	0.6	4.2	0.5	4.2	0.5	3.8	0.9	2.1^b^	0.3	2.0	0.3
Therapeutic dose	4.3	0.6	4.3	0.6	4.2	0.7	4.3	0.6	4.2	0.6	4.3	0.6	4.2	0.6

^a^Mean doses when the dose reduction was completed in the 4-week group

^b^Mean doses when the dose reduction was completed in the 26-week group

## Discussion

This study comprises the first investigation to use a mixed model approach to analyze PRS and determine the course of manifestation over the entire study period. Several conclusions may be drawn from this prospective, multicenter study in schizophrenia patients treated with risperidone.

In this study, male or female, age at onset and PANSS total scores were associated with PRS. Female sex was associated with severity of PRS, which were positively linked to the PANSS total scores and negatively correlated with age at onset. Most previous studies have been conducted on hyperprolactinemia risk factors. The results of a number of studies have suggested that higher antipsychotic doses, a longer duration of treatment, female sex, younger age, stress, and combination treatment with an antidepressant comprised risk factors for hyperprolactinemia [15, 20]. In a multiple logistic regression, younger age was an independent risk factor of hyperprolactinemia [6]. Several studies have also demonstrated that the risk of hyperprolactinemia decreased with age [6, 31]. We did not identify an association between PRS and age; however, a negative association was identified between PRS and age at onset. Similarly, it has been demonstrated that the mean plasma prolactin level was increased in early-onset schizophrenia spectrum psychosis patients compared with no-early-onset patients [32]. Another finding of this study is that the risperidone dose was positively associated with the severity of PRS, which is consistent with the previous study. Furthermore, it has been suggested that a dose-response relationship is uncertain [33]. Previous evidence indicates there was no correlation between the risperidone dose and the incidence of side effects presumed to be caused by hyperprolactinemia in patients [34]. Risperidone dose may be more related to PRS than hormones. However, few studies have investigated the relationship between PRS and the PANSS scores. In a Korean study, the prolactin levels in acute psychiatric inpatients who received risperidone were not significantly correlated with improvements in the total BPRS scores [35].

As the treatment duration increased, the prevalence of PRS decreased, even in the dose-reduction groups, which may be attributed to the tolerability of the drug, as well as the dropouts. However, the dose-reduction strategies did not have a significant influence on the descending trend in PRS occurrence, even at a low dose, and the PRS remained similar to the relatively high dose. This finding was supported by a review in which the authors concluded that following long term administration of antipsychotics, such as risperidone, tolerance and decreases in the prolactin level may occur [4].

Several limitations should be considered in the interpretation of these results. One limitation is that the patients and clinicians were not blind to the treatment group. Thus, bias as a result of expectation is a possibility that cannot be ruled out. Another limitation is that we only reassessed the PRS of patients who continued treatment in the study. This approach was implemented because intolerable side effects, relapse, and other reasons resulted in the discontinuation of treatment in a proportion of the patients. Moreover, we used a binary variable that was somewhat limited to describe PRS, which does not reflect the severity of PRS. Finally, we only assessed the PRS; we did not measure the plasma prolactin level or other hormones with potential interactions. The assessment of patients only based on symptoms related to increased plasma prolactin levels is not ideal. Thus, further research is required to clarify this complex situation to assess the potential correlations between abnormal laboratory values and the clinical manifestations of hyperprolactinemia.

## Conclusions

In summary, the PRS severity was alleviated during the one year treatment period because of the dose reduction. Attention should focus on the side effects of hyperprolactinemia during long-term treatment, especially with a high dose, females, younger age at onset, and more severe patients, because hyperprolactinemia related side effects may comprise important concerns in patients undergoing chronic therapy. Furthermore, a mixed model approach may be implemented to assess PRS in longitudinal studies with multiple time points.
